# Effectiveness of interventions for caregiver burden and quality of life of informal caregivers of stroke survivors: a systematic review and meta-analysis

**DOI:** 10.3389/fpubh.2026.1932478

**Published:** 2026-10-07

**Authors:** Meijuan Lin, Sicong Xie, Benguo Wang, Alice Kwai-Yee Siu

**Affiliations:** 1 School of Medicine, The Chinese University of Hong Kong, Shenzhen, China; 2 Department of Neurology, The Second Affiliated Hospital of The Chinese University of Hong Kong, Shenzhen, China

**Keywords:** caregiver burden, informal caregivers, quality of life, stroke survivor, systematic review and meta-analysis

## Abstract

**Background:**

Informal caregivers, who play a pivotal role in post-stroke care, frequently report substantial caregiver burden and reduced quality of life (QoL), which may adversely affect caregiving capacity and stroke rehabilitation outcomes. However, evidence regarding effective interventions to lessen caregiver burden and improve the QoL of informal caregivers remains fragmented.

**Aims:**

This study aimed to synthesize evidence on the effectiveness of interventions for reducing the caregiver burden and improving the QoL of informal caregivers of stroke survivors.

**Methods:**

A systematic search of eight databases (PubMed, MEDLINE, Embase, Cochrane Library, Scopus, Web of Science, CINAHL, and PsycINFO) was conducted until June 18, 2026. Randomized controlled trials (RCTs) published in English were included if they investigated the caregiver burden or QoL of informal caregivers of stroke survivors. The revised Cochrane risk-of-bias tool (RoB 2.0) was used to assess the risk of bias in the eligible studies. Random-effects meta-analyses using the DerSimonian-Laird method were conducted to pool data on caregiver burden and QoL. Subgroup analyses were performed based on the defined intervention characteristics.

**Results:**

Overall, 39 RCTs involving 3,096 informal caregivers of stroke survivors were included in this analysis. Meta-analyses showed that standalone (psycho)education yielded the largest effect size in reducing the caregiver burden (*k* = 12; Hedges’ g = 0.87; 95% confidence interval [CI]: 0.52 to 1.23; *I^2^* = 86.31%), followed by the medium effect size of (psycho)education plus skill building (*k* = 12; Hedges’ g = 0.62; 95% CI: 0.28 to 0.97; *I^2^* = 84.86%) and the small effect size of multicomponent interventions (the combination of (psycho)education, skill building, and support interventions; *k* = 3; Hedges’ g = 0.27; 95% CI: 0.03 to 0.52; *I^2^* = 0.00%). However, the combination of (psycho)education and support interventions was not significantly associated with caregiver burden (*k* = 4; Hedges’ g = 0.23; 95% CI: −0.04 to 0.49; *I^2^* = 0.00%). Standalone (psycho)education was the only intervention associated with significant improvements in the QoL of informal caregivers of stroke survivors (*k* = 7; Hedges’ g = 1.11; 95% CI: 0.39 to 1.83; *I^2^* = 94.09%). Subgroup analyses indicated that larger effect sizes for both caregiver burden and QoL tended to be observed when interventions exclusively targeted informal caregivers, were researcher-led, spanned both hospital and home settings, used hybrid delivery modalities (face-to-face and remote), and comprised 5*–*9 sessions.

**Conclusion:**

Standalone (psycho)education demonstrated the largest pooled effect estimate for reducing caregiver burden and improving the QoL of informal caregivers of stroke survivors. These findings hold important clinical and practical implications for healthcare professionals by informing the design and implementation of caregiver-support interventions. Further high-quality studies are warranted to confirm these findings and identify the optimal intervention package.

**Systematic review registration:**

https://www.crd.york.ac.uk/PROSPERO/register/TemplatePreview, identifier: CRD420251152425.

## 1 Introduction

Stroke is a leading cause of disability and mortality worldwide, accounting for 160.5 million disability-adjusted life-years and 7.3 million deaths globally in 2021 (1). Approximately 50–70% of stroke survivors experience persistent physical, cognitive, or psychological impairments (2). Moreover, the risk of recurrent stroke remains considerable after the initial attack, with the recurrence rates being 10.4 and 16.1% at 1 and 2 years, respectively (3, 4). Stroke places a substantial burden on healthcare systems and society because of the long-term care dependency and ongoing rehabilitation needs of patients. Over the past two decades, family-centered care has been increasingly advocated in stroke rehabilitation alongside a shift in post-stroke management toward community-based care models (5, 6). This change highlights the pivotal role played by family caregivers throughout the post-stroke care continuum. It is estimated that up to 80% of patients discharged after initial stroke hospitalization depend on informal caregivers for assistance with activities of daily life (5).

Informal caregivers are typically family members or friends who provide unpaid care to stroke survivors (7). Despite often lacking adequate preparation and caregiving knowledge, they are required to devote substantial time to caregiving and assume multiple roles in supporting survivors’ daily activities and rehabilitation (8, 9). The intensive demands of caregiving have been associated with their negative physical and psychological health. For example, evidence shows that caregiver burden affects 67% of informal caregivers and tends to increase over time, rising from 37.5% at 6 months to 47.5% at 10 years (10, 11). Informal caregivers have also reported a poorer quality of life (QoL) compared to that of the general population (12). Excessive caregiver burden and diminished QoL may compromise the continuity and quality of post-stroke care, thereby adversely affecting patient rehabilitation outcomes and prognosis (13–15). Moreover, some informal caregivers may seek healthcare services for their own health-related problems, further exacerbating the pressure on healthcare systems. These intertwined challenges underscore the need for proactive interventions to support informal caregivers during poststroke care.

The World Stroke Organization, in its service guidelines and action plans, has emphasized the importance of providing long-term education and support to informal caregivers of stroke survivors (16). However, despite an increasing body of research evaluating interventions to reduce the caregiver burden and improve the QoL of informal caregivers, the evidence remains inconsistent. For example, one meta-analysis of 17 studies showed that (psycho)education was associated with significant reductions in caregiver burden (17), whereas another found no significant effects of (psycho)education on caregiver burden or the QoL of stroke caregivers (18). In addition, although existing systematic reviews have evaluated specific populations (e.g., caregivers or stroke survivor-caregiver dyads) (18–20), intervention types (e.g., (psycho)education or psychosocial interventions) (18, 19, 21), or delivery modalities (e.g., remote or face-to-face) (17, 19, 21–23), several critical knowledge gaps remain. First, because prior reviews restricted their scope to narrow intervention types or delivery modalities, no comprehensive synthesis has compared the relative effectiveness of various intervention categories based on the full body of randomized controlled trial (RCT) evidence. Second, key intervention and implementation features (e.g., duration and session frequency) have rarely been systematically examined to determine the optimal interventions for lessening caregiver burden and enhancing caregivers’ QoL. Consequently, evidence-based, specific guidance on how to support stroke caregivers remains limited. To address these gaps, this systematic review and meta-analysis aimed to comprehensively evaluate and compare the effectiveness of different intervention strategies in reducing caregiver burden and improving informal stroke caregivers’ QoL, and to examine key intervention characteristics, including dosage, associated with meaningful outcomes.

## 2 Methods

### 2.1 Search strategy

An initial comprehensive search of the following eight electronic databases was performed from their inception until August 14, 2025 (updated on June 18, 2026): PubMed, MEDLINE, Embase, Web of Science, the Cochrane Library, Scopus, PsycINFO, and CINAHL. The search strategy combined Medical Subject Heading terms and relevant keywords by using Boolean operators, covering five key concepts: stroke, caregivers, caregiver burden, quality of life, and randomized controlled trials (RCTs). The details are presented in Supplementary Table S1.

### 2.2 Inclusion and exclusion criteria

The eligibility criteria were outlined using the population, intervention, comparator, outcome, and study design (PICOST) framework (24). The inclusion criteria were as follows. (1) RCTs or cluster RCTs were included, as well as (2) studies focusing on informal caregivers of stroke survivors at any stage across the post-stroke continuum. Informal caregivers were defined as family members who administered primary care to stroke survivors in their daily lives without payment (17, 25). Furthermore, studies without restrictions pertaining to the ages of informal caregivers or stroke survivors were included, as well as (3) those on intervention strategies either on caregivers or on dyads were considered eligible, irrespective of whether the interventions were conducted in hospital or a community-dwelling setting; (4) with control conditions including usual care, waitlist control, or any passive interventions; and (5) in which outcomes of interest were caregiver burden and QoL, regardless of whether they were primary or secondary outcomes. The exclusion criteria were as follows: (1) studies examining interventions targeting stroke survivors rather than caregivers or dyads; (2) studies in which baseline and postintervention data were unavailable; (3) studies for which articles were in languages other than English; and (4) reviews, trial registries, conference abstracts, letters, protocols, notes, and books. The detailed eligibility criteria are outlined in Supplementary Table S2.

### 2.3 Study selection

All the searched records were imported into EndNote 20 (Clarivate) to remove duplicates. Two reviewers then independently screened the titles and abstracts of the remaining articles. Next, potentially relevant studies were subjected to full-text retrieval and eligibility assessment. Finally, the two assessors cross-checked their reports; any discrepancies were resolved by a third reviewer. The reference lists of the selected studies and topic-related reviews were manually screened to avoid missing eligible articles.

### 2.4 Data extraction

Two authors independently extracted data from the included studies by using a standardized data extraction form. The following information was extracted from each study: author, country, publication year, study design, sample size, caregivers’ age, intervention characteristics (theoretical framework, intervention design, target population, intervention implementer and site, intervention delivery format and modality, number of sessions, and duration), control group, timing of data collection, caregiver burden and QoL outcomes, and measurement scales. Interventions were categorized into three types adapted from prior reviews (26, 27): (psycho)education, skill building, and support interventions. (psycho)education was defined as interventions involving educational content or psychotherapeutic strategies (e.g., information about stroke, medication, available resources, counseling, and cognitive behavioral therapy). Skill building was defined as interventions that equip caregivers with practical caregiving skills (e.g., problem solving, caregiving techniques, time management, goal setting, and communication skills). Finally, support interventions were defined as structured support provided by researchers, peers, or health professionals (e.g., support groups, discussion forums, or referral services). Two reviewers independently classified all interventions, resolving discrepancies through discussion or senior-reviewer adjudication. RCTs with more than two arms were combined using the formulas described in the Cochrane Handbook (28). When an outcome of interest was measured using multiple scales, data from the most commonly used scales were extracted. Overall QoL scores were prioritized for data analyses; physical and mental health scores were synthesized for the total QoL scores.

### 2.5 Quality appraisal

Two reviewers independently assessed the methodological quality of the included studies by using the Cochrane Risk of Bias Tool version 2 (RoB 2.0) (29, 30). Using this tool, the ratings of low risk, some concerns, and high risk were assigned for RCTs according to the randomization process, deviations from the intended intervention, missing outcome data, outcome measurements, and selection of reported results (29). For cluster RCTs, an additional domain related to the timing of participant identification and recruitment was also considered in the RoB 2.0 assessment (30).

### 2.6 Statistical analysis

Initial analyses involved calculation of the pre-post change scores and their standard deviations (SDs) for continuous outcomes. When change SDs were not directly reported in original studies, they were calculated using the standard formula below, assuming the correlation coefficient of *Corr* = 0.5 in accordance with the Cochrane Handbook (31):

$$SD_{\text{change}}=\sqrt{SD_{\text{baseline}}^{2}+SD_{\text{post}}^{2}-2\times \text{Corr}\times SD_{\text{baseline}}\times SD_{\text{post}}}$$

For caregiver burden, change scores were calculated by subtracting post-intervention means from baseline, whereas for QoL, baseline means were subtracted from post-intervention values to ensure that positive scores consistently represented beneficial effects. Standardized mean differences were calculated using Hedges’ g to pool effect sizes by using random-effects models based on the DerSimonian-Laird method. Heterogeneity was quantified using the *I^2^* statistic, with *I^2^* > 75% indicating considerable heterogeneity (32). Subgroup analyses were performed to explore the associations between the intervention characteristics and outcomes. For each meta-analysis of 10 or more studies, publication bias was evaluated visually by using funnel plots and quantitatively by using Egger’s test (33). To evaluate the robustness of the primary estimates, sensitivity analyses were systematically conducted. First, a leave-one-out sensitivity analysis was performed to examine the influence of individual studies on the pooled estimates. Second, to address potential unit-of-analysis bias from the single included cluster RCT (72), a sensitivity analysis was performed by inflating its standard error using a design effect (Design Effect = 1 + (M – 1) × ICC, assuming ICC = 0.05), in accordance with the Cochrane Handbook (28). Finally, an additional sensitivity analysis was conducted by excluding studies rated as having high risk of bias to assess the stability of the overall findings. Meta-regression analyses were conducted to determine potential sources of heterogeneity when at least 10 studies were included in the meta-analysis. The mean caregiver age and intervention duration were included as covariates in the meta-regression analyses. All statistical analyses were completed using Stata (MP version 17.0), and statistical significance was defined as a two-sided *p*-value of < 0.05.

## 3 Results

### 3.1 Search results

Of the 5,810 records identified from the database search, 119 remained after the removal of duplicates and title and abstract screening. After the full-text screening, 80 studies were excluded from this review. Finally, 39 studies published in English between 2003 and 2025 met the inclusion criteria (Figure 1).

**Figure 1:**
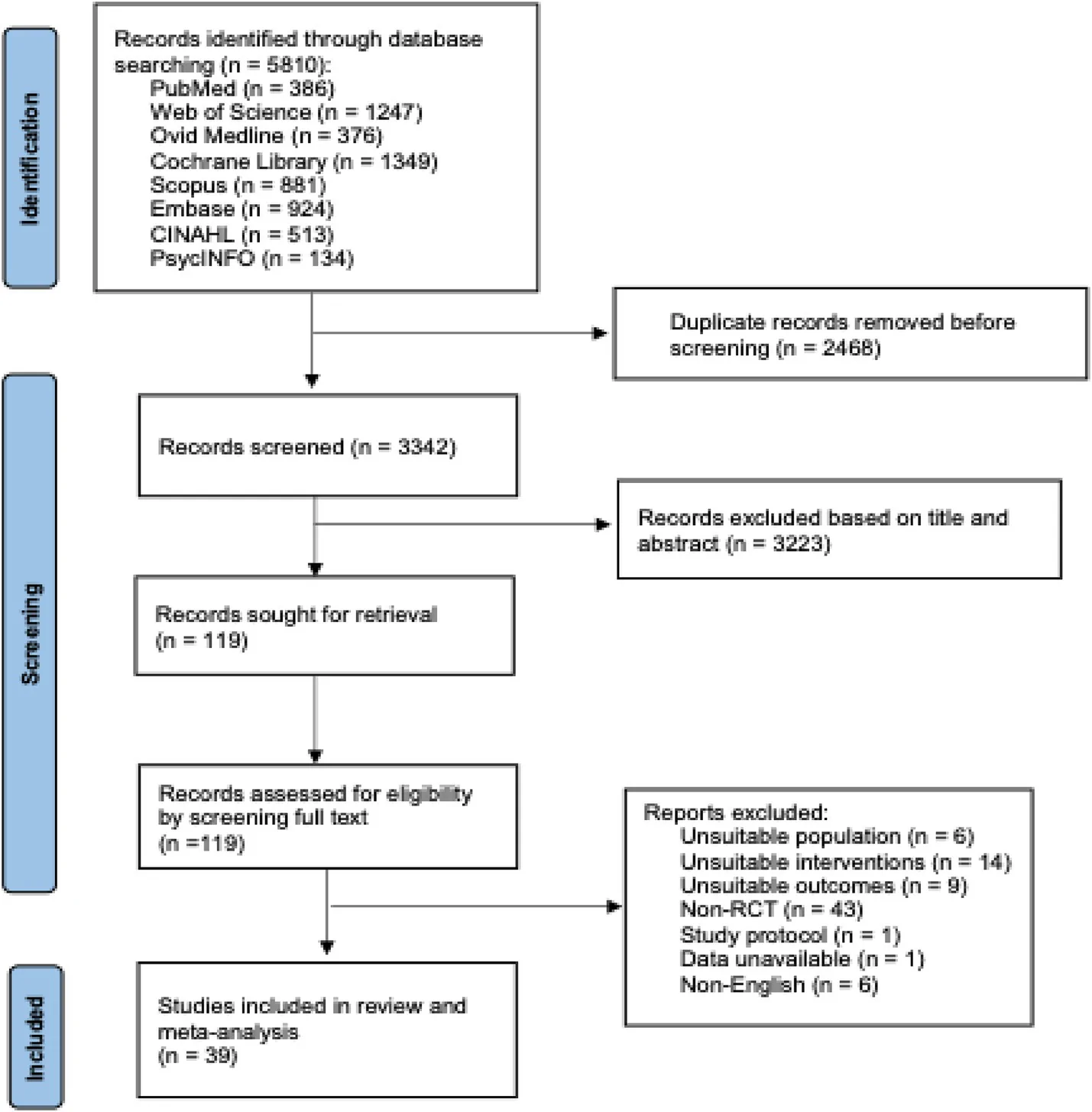
PRISMA flow of study selection.

### 3.2 Characteristics of studies and participants

In total, 3,096 participants were included across the 39 studies. The median sample size was 72 (range: 17*–*210), and the mean participant age was 52.53 (range: 21.89–70.00) years. The percentage of female informal caregivers ranged from 33.64 to 92.59%, with a median of 73.10%. All of the included studies were individual RCTs (34–71), barring one, which was a cluster RCT (72). Ten studies were conducted in Iran (39, 41, 42, 45, 46, 53, 54, 60, 63, 68); seven in China (35, 38, 43, 44, 48, 52, 57); five in Turkey (34, 37, 65, 67, 70); four each in Brazil (61, 62, 64, 66) and the United States of America (40, 50, 55, 71); three in Australia (47, 49, 59); and one each in Spain (56), Thailand (72), Egypt (58), the United Kingdom (36), Sweden (51), and the Netherlands (69). Caregiver burden was assessed in 23 studies (34, 35, 39, 42–44, 48–50, 52, 53, 55, 56, 59, 60, 62–65, 68, 69, 71, 72), QoL of informal caregivers was evaluated in seven studies (38, 41, 45, 46, 51, 66, 67), and both outcomes were assessed in nine studies (36, 37, 40, 47, 54, 57, 58, 61, 70). The Zarit Burden Interview was the most used to measure caregiver burden, while the WHOQOL-BREF and Short Form-36 Health Survey were most frequently employed to evaluate QoL. The details of the included studies are presented in Table 1.

**Table 1:** Characteristics of the included studies and participants.

Source (year, Country)	Study design	Sample size *N* (IG/CG)	Age (mean (SD), year) (IG/CG)	Gender (female, %)	Intervention category	Intervention description	Duration	Control group	Data collection	Outcomes ^#^	Theoretical framework
Baykal (2022, Turkey) (67)	RCT	60 (IG1: 20, IG2: 20, CG: 20)	IG1: 42.35 ± 13.10 IG2: 45.16 ± 14.89 CG: 47.55 ± 11.56	77.97%	(Psycho)education + support interventions	Discharge training via a webpage (IG1) Discharge training via a booklet (IG2)	3 months	Usual care	Baseline, 3 months	SF-12: PCS ㊉ MCS ㊉	NA
Bierhals (2023, Brazil) (66)	RCT	48 (24/24)	53.38 ± 11.91/53.54 ± 14.05	87.50%	(Psycho)education + support interventions	Three home visits by nurses 1 month after hospital discharge to provide stroke-related education and emotional support	1 month	Usual care	Baseline and 2 and 12 months	WHOQOL-BREF ㊀: PCS ㊀ MCS ㊀	NA
Bitek (2023, Turkey) (65)	RCT	80 (40/40)	51.60 ± 14.18/54.61 ± 11.73	71.01%	(Psycho)education	Discharge training, telephone counseling service	3 months	Standard care	Baseline, 3 months	ZBI ㊉	NA
Costa (2025, Brazil) (64)	RCT	58 (29/29)	52.10 ± 13.30/51.40 ± 11.60	79.31%	(Psycho)education	Online education sessions, telephone counseling service	3 months	Usual care	Baseline and 1, 2, and 3 months	CBS ㊀	NA
Dahmardeh (2025, Iran) (63)	RCT	60 (30/30)	22.12 ± 0.85/21.89 ± 0.87	54.24%	(Psycho)education + skill-building	Six supportive training sessions	1 month	Routine hospital instructions	Baseline, 3 months	CBI ㊉	NA
Day (2021, Brazil) (62)	RCT	48 (24/24)	53.38 ± 11.91/53.54 ± 14.05	87.50%	(Psycho)education + support interventions	A systematic follow-up intervention	1 month	Usual care	Baseline and 2 and 12 months	CBS ㊀	NA
Deepradit (2023, Thailand) (72)	cluster RCT	62 (31/31)	54.16 ± 15.19/51.35 ± 18.09	74.19%	(Psycho)education + skill-building + support interventions	A stressor assessment and received the family-based program	10 weeks	Usual care	Baseline and 4 and 12 weeks	ZBI ㊉	Neuman System Model
Deyhoul (2020, Iran) (60)	RCT	90 (45/45)	41.10 ± 11.00/40.60 ± 11.70	64.44%	(Psycho)education	A family-focused dyadic psychoeducational intervention	2 weeks	Usual care	Baseline, 2 weeks, 2 months	CBI ㊉	Family-centered empowerment model
Eames (2013, Australia) (60)	RCT	61 (31/30)	NA	54.35%	(Psycho)education + support interventions	Educational sessions, support package	3 months	Usual care	Baseline, 3 months	CSI ㊀	Health Belief Model, adult learning principles
Elsheikh (2022, Egypt) (58)	RCT	110 (55/55)	35 (25–55)/35 (25–57)	74.55%	(Psycho)education + skill-building + support interventions	A tailored multidimensional Intervention (psychoeducation, skill building, peer support)	6 months	Simple educational instructions	Baseline and 3 and 6 months	sZBI ㊀ WHOQOL-BREF ㊀	Evidence-based conceptual framework
Farahani (2021, Iran) (68)	RCT	120 (60/60)	43.98 ± 13.80/43.41 ± 11.25	71.55%	(Psycho)education	Supportive home care program (eight educational sessions)	2 weeks	Routine hospital education	Baseline, 2 weeks	CBI ㊉	NA
Fu (2022, China) (58)	RCT	68 (34/34)	68.56 ± 5.90/67.00 ± 4.42	47.06%	(Psycho)education + skill-building	Benefit-finding intervention program	9 weeks	Routine health education	Baseline, 9 weeks	ZBI ㊉ AC-QoL ㊉	Stress-coping theory, cognitive adaptation theory
García-Pérez (2024, Spain) (56)	RCT	55 (28/27)	NA	Not reported	(Psycho)education + skill-building	Early occupational therapy intervention, coping strategies	1 month	Standard care	Baseline, 3 months	CBS ㊉	NA
Hartke (2003, USA) (55)	RCT	88 (43/45)	69.74 ± 5.39/69.69 ± 6.59	76.14%	(Psycho)education + support interventions	An eight-session psychoeducational telephone group	8 weeks	Usual care	Baseline, 8 weeks, and 6 months	ZBI ㊀	Stress and coping model
Hekmatpou (2019, Iran) (54)	RCT	100 (50/50)	48.52/45.14	63.00%	(Psycho)education	Education, counseling	2 weeks	Routine training	Baseline, 1 month	ZBI ㊉ SF-36 ㊉: PCS ㊀ MCS ㊉	NA
Hosseini (2022, Iran) (53)	RCT	72 (36/36)	44.11 ± 12.524/44.47 ± 10.840	91.67%	(Psycho)education	Training and counseling	4 weeks	Usual training	Baseline, 4 weeks	CBI ㊉	NA
Jia (2024, China) (52)	RCT	80 (40/40)	50.00 ± 10.50/49.50 ± 10.75	76.25%	(Psycho)education + skill-building + support interventions	Informational, emotional, instrumental, and appraisal support combined with trajectory-based support conducted via the WeChat platform	9 weeks	Usual care	Baseline, 9 weeks	ZBI ㊉	The Corbin and Strauss chronic illness trajectory framework, the concept of social support
Kootker (2019, Netherlands) (70)	RCT	50 (23/27)	59.80 ± 12.50/59.60 ± 10.20	64.00%	(Psycho)education + skill-building	Cognitive behavioral therapy augmented with movement or occupational therapy	4 months	Computerized cognitive training	Baseline and 4, 8, and 12 months	CSI ㊀	Cognitive behavioral therapy
Larson (2005, Sweden) (51)	RCT	100 (50/50)	68.16 ± 10.42/66.66 ± 9.82	80.00%	(Psycho)education + support interventions	A nurse-led support and education program	6 months	Usual care	Baseline and 6 and 12 months	A visual analog scale ㊀	NA
LeLaurin (2021, USA) (50)	RCT	40 (13/27)	60.30 ± 10.10 (total)	92.45%	(Psycho)education + skill-building	A support and problem-solving intervention	8 weeks	Attention control Standard care	Baseline and 9 and 25 weeks	sZBI ㊀	D’Zurilla and Nezu’s relational/problem-solving model of stress, Lazarus’ stress appraisal and coping theory
Lopez (2025, USA) (71)	RCT	210 (105/105)	60.00 ± 12.00/59.00 ± 11.00	88.10%	(Psycho)education + skill-building	A telephone- and online-based problem-solving intervention	8 weeks	Usual care	Baseline and 9 and 21 weeks	ZBI ㊉	Relational/problem-solving model of stress
Marsden (2010, Australia) (49)	RCT	17 (9/8)	66.30 ± 10.10/69.60 ± 11.50	88.24%	(Psycho)education + support interventions	Physical activity, education, self-management principles, a “healthy options” morning tea	7 weeks	Waiting list	Baseline and 7 and 21 weeks	CSI ㊀	NA
Mei (2018, China) (48)	RCT	75 (IG1: 25, IG2: 22, CG: 28)	IG1: 69.67 ± 2.35 IG2: 70.00 ± 2.00 CG: 69.80 ± 5.58	72.00%	(Psycho)education + skill-building	IG1: Modified 8-week reminiscence therapy (Dyads received intervention) IG2: Modified 8-week reminiscence therapy (Caregivers received intervention)	2 months	Routine health education	Baseline and 2, 3, and 5 months	CBI ㊉	NA
Minshall (2020, Australia) (47)	RCT	64 (35/29)	65.00 ± 13.40/61.00 ± 14.00	71.88%	(Psycho)education + skill-building	A personalized psychosocial intervention	3 months	Usual care	Baseline and 3, 6, and 12 months	mCSI ㊀ EQ-5D-5L ㊉	A collaborative therapy framework
Mohammadi (2023, Iran) (46)	RCT	84 (42/42)	46.20 ± 12.20/46.10 ± 10.50	65.82%	(Psycho)education	An educational intervention	12 weeks	Usual care	Baseline, 12 weeks	SF-36 ㊉: PCS ㊀ MCS ㊉	NA
Moreira (2018, Brazil) (61)	RCT	33 (IG1: 11, IG2: 11, CG: 11)	IG1: 55.09 ± 7.73 IG2: 55.91 ± 14.52 CG: 52.91 ± 17.07	81.82%	(Psycho)education + skill-building	Postural hygiene training and kinesiotherapy (IG1), adaptation of the home environment (IG2)	12 weeks	Standard intervention	Baseline and 12 and 24 weeks	PSS ㊀ WHOQOL-BREF ㊉	NA
Monjezi (2025, Iran) (45)	RCT	104 (52/52)	39.08 ± 10.45/42.08 ± 10.97	43.88%	(Psycho)education	Four education sessions and telephone follow-ups	6 weeks	Routine hospital education	Baseline, 2 months	AC-QoL ㊉	Roy’s adaptation model
Mou (2022, China) (44)	RCT	40 (20/20)	45.61 ± 12.14/48.10 ± 12.20	47.50%	(Psycho)education	A family-focused dyadic psychoeducational intervention	4 weeks	Usual care	Baseline, 4 weeks	CBI ㊉	Double ABC-X model
Mou (2023, China) (43)	RCT	162 (81/81)	48.38 ± 11.77/51.07 ± 11.76	54.94%	(Psycho)education	A family-focused dyadic psychoeducational intervention	4 weeks	Usual care	Baseline, 4 weeks, 3 months	CBI ㊉	Double ABC-X model
Nazari (2024, Iran) (42)	RCT	64 (32/32)	51.56 ± 10.42/53.60 ± 9.03	55.00%	(Psycho)education	Fifteen online education sessions	1.5 months	Standard hospital education	Baseline and 1.5, and 2 months	ZBI ㊉	Kern’s model
Nooreddini (2024, Iran) (41)	RCT	80 (40/40)	NA	51.25%	(Psycho)education	Self-care support program	4 weeks	Usual care	Baseline, 2 months	SF-12 ㊉	Orem’s model
Ostwald (2014, USA) (40)	RCT	159 (80/79)	63.61 ± 11.02/61.34 ± 9.77	74.84%	(Psycho)education + skill-building	Home-based psychoeducational and mailed information programs	6 months	Mailed information	Baseline and 3, 6, 9, and 12 months	ZBI ㊀ SF-36 ㊀	Double ABC-X model
Sarmadi (2025, Iran) (39)	RCT	110 (55/55)	39.64 ± 12.51/45.60 ± 10.92	69.00%	(Psycho)education	Education based on the seven-step (7E) learning cycle	8 weeks	Routine education	Baseline, 8 weeks	CBI ㊉	Seven-step learning cycle model
Tanrikulu (2024, Turkey) (70)	RCT	40 (20/20)	52.15 ± 10.70/53.07 ± 14.90	92.59%	(Psycho)education	A family support program based on a nurse-led case management model	6 months	Routine health care and follow-up	Baseline and 3 and 6 months	ZBI: ㊉ WHOQOL-BREF: ㊉	Nurse-Led Case Management Model
Tsai (2024, China) (38)	RCT	90 (45/45)	49.28 ± 10.79/49.90 ± 12.68	64.63%	(Psycho)education	A nurse-led therapeutic conversations intervention	4 weeks	Usual care	Baseline, 1 month	SF-12: PCS ㊀ MCS ㊉	NA
Ugur (2019, Turkey) (37)	RCT	104 (52/52)	51.20 ± 13.45/53.25 ± 12.70	91.86%	(Psycho)education	Training and consultancy	10 weeks	Usual care	Baseline, 10 weeks	CBS: ㊉ Sf-36: ㊀ MCS ㊉	Model of daily living activities
Walker (2020, UK) (36)	RCT	35 (18/17)	63.33 ± 12.72/61.88 ± 13.36	74.29%	(Psycho)education + skill-building	A biopsychosocial intervention	6 weeks	Usual care	Baseline, 6 months	CBS ㊀ EQ-5D-5L ㊉	NA
Wang (2021, China) (35)	RCT	110 (55/55)	47.30 ± 10.40/47.60 ± 10.20	33.64%	(Psycho)education + skill-building	Education and muscle relaxation program	12 months	Common rehabilitation advice	Baseline and 3, 6, and 12 months	ZBI ㊉	NA
Yilmaz (2019, Turkey) (34)	RCT	65 (33/32)	47.43 ± 11.29/53.43 ± 13.51	84.09%	Others	Progressive muscle relaxation	8 weeks	Blank control	Baseline, 8 weeks	ZBI ㊀	NA

IG, intervention group; CG, control group; CBI, Caregiver Burden Inventory; ZBI, Zarit Burden Interview; CBS, Caregiver Burden Scale; CSI, Caregiver Strain Index; sZBI, short-version Zarit Burden Interview; mCSI, Modified Caregiver Strain Index; PSS, Perceived Stress Scale; SF-36, Short Form-36 Health Survey; SF-12, 12-item Short Form survey; AC-QoL, Adult Carer Quality of Life questionnaire; WHOQOL-BREF, the World Health Organization’s Quality of Life assessment (26-item); EQ-5D-5L, European Quality of Life-5-Dimensions; PCS, physical component summary for QoL; MCS, mental component summary for QoL; ^#^Reported the postintervention comparison outcomes between the control and intervention groups.

### 3.3 Intervention characteristics

Over half of the studies were based on theoretical frameworks (37, 39–45, 47, 50, 52, 55, 57–60, 69–72), while the others were not theory-based (34–36, 38, 46, 48, 49, 51, 53, 54, 56, 61–68). Twenty-one of the studies focused on dyadic interventions (37–44, 47–49, 53, 56, 59, 60, 62, 64, 66, 67, 69, 72) rather than on informal caregivers alone (34–36, 45, 46, 50–52, 54, 55, 57, 58, 61, 63, 65, 68, 70, 71). Interventions were primarily delivered by healthcare professionals (36, 38, 41, 43, 44, 47, 48, 50, 51, 53, 56, 58, 59, 61, 62, 66, 69), followed by researchers (34, 35, 37, 42, 45, 46, 52, 54, 60, 63, 65, 67, 68, 71, 72) and multidisciplinary teams (39, 40, 49, 55, 57, 64, 70). Twenty studies tailored interventions to individuals (38, 40, 41, 45, 47, 50, 53, 56–59, 61–63, 66, 67, 69–72), 10 employed a one-size-fits-all approach (34–36, 42, 48, 49, 51, 60, 65, 68), and nine used mixed approaches (37, 39, 43, 44, 46, 52, 54, 55, 64). Sixteen studies used (psycho)education as the sole intervention (37–39, 41–46, 53, 54, 60, 64, 65, 68, 70), 12 adopted (psycho)education with skill-building (35, 36, 40, 47, 48, 50, 56, 57, 61, 63, 69, 71), seven combined (psycho)education with support (49, 51, 55, 59, 62, 66, 67) and three incorporated all three intervention types (multicomponent interventions) (52, 58, 72). Progressive muscle relaxation was used as an intervention in one study (34). The most common modalities of intervention delivery were face-to-face (34–38, 40, 48, 49, 51, 57, 60–63, 66, 68) and hybrid modalities (39, 41, 43–45, 47, 54, 56, 58, 59, 65, 67, 69, 70, 72), whereas remote delivery was used in only eight studies (42, 46, 50, 52, 53, 55, 64, 71). Interventions in 22 studies were implemented individually (34, 37, 38, 40, 41, 45, 47, 48, 50, 53, 56, 57, 59, 61–64, 66, 67, 69–72); in five studies, interventions were implemented in group settings (42, 49, 51, 55, 60), and in 12 studies, intervention involved a combination of individual and group-based formats (35, 36, 39, 43, 44, 46, 52, 54, 58, 65, 68). In 17 studies, interventions were provided at participants’ homes (34, 36, 37, 40, 42, 46, 48, 50, 53, 55, 57, 58, 62, 64, 66, 71, 72); in eight studies, they were administered during hospitalization (38, 45, 49, 51, 60, 61, 67, 69) and in 14 studies, interventions were made available in both settings (35, 39, 41, 43, 44, 47, 52, 54, 56, 59, 63, 65, 68, 70). The intervention duration typically ranged from 2 to 6 months (34, 37, 39, 40, 46–48, 50–52, 55, 57–59, 61, 64, 65, 67, 69–72), with 16 studies reporting durations of less than 2 months (36, 38, 41–45, 49, 53, 54, 56, 60, 62, 63, 66, 68); intervention exceeded 6 months in only one study (36). Most studies employed 5–9 intervention sessions (36, 37, 39, 43, 44, 47–52, 54–57, 59, 63–65, 70–72), with nine studies reporting ≥10 sessions (34, 35, 40, 42, 46, 58, 61, 68, 69) and eight studies reporting ≤4 sessions (38, 41, 45, 53, 60, 62, 66, 67). More than 90% of the studies used usual care as the comparator intervention (35–48, 50–68, 70–72).

### 3.4 Risk of bias assessment

Of the 38 individual RCTs, 28 were rated as having an overall low risk of bias (34–36, 38, 40, 42–53, 56–59, 61, 62, 64, 66–69, 71), one as raising some concerns (70) and nine as having a high risk of bias (34, 37, 39, 41, 54, 55, 60, 63, 65). In particular, inadequate concealment of the randomization process was identified in five studies (34, 37, 41, 55, 63), missing outcome data were recognized in two studies (54, 70), potential bias of outcome measurement was identified in three studies (39, 60, 65), and selected outcome reporting was recognized in two studies (54, 63). Regarding the remaining cluster RCT, it was rated as raising some concerns owing to the potential bias arising from the randomization process (Supplementary Figure S1) (72).

### 3.5 Meta-analyses

#### 3.5.1 Caregiver burden

Meta-analyses of 31 studies showed that standalone (psycho)education yielded the largest effect in terms of reducing caregiver burden (*k* = 12; Hedges’ g = 0.87; 95% CI: 0.52 to 1.23; *I^2^* = 86.31%), followed by (psycho)education plus skill building (*k* = 12; Hedges’ g = 0.62; 95% CI: 0.28 to 0.97; *I^2^* = 84.86%) and multicomponent interventions (combination of (psycho)education, skill building, and support interventions) (*k* = 3; Hedges’ g = 0.27; 95% CI: 0.03 to 0.52; *I^2^* = 0.00%). However, no significant association was observed between caregiver burden and interventions combining (psycho)education with support (*k* = 4; Hedges’ g = 0.23; 95% CI: −0.04 to 0.49; *I^2^* = 0.00%) (Figure 2).

**Figure 2:**
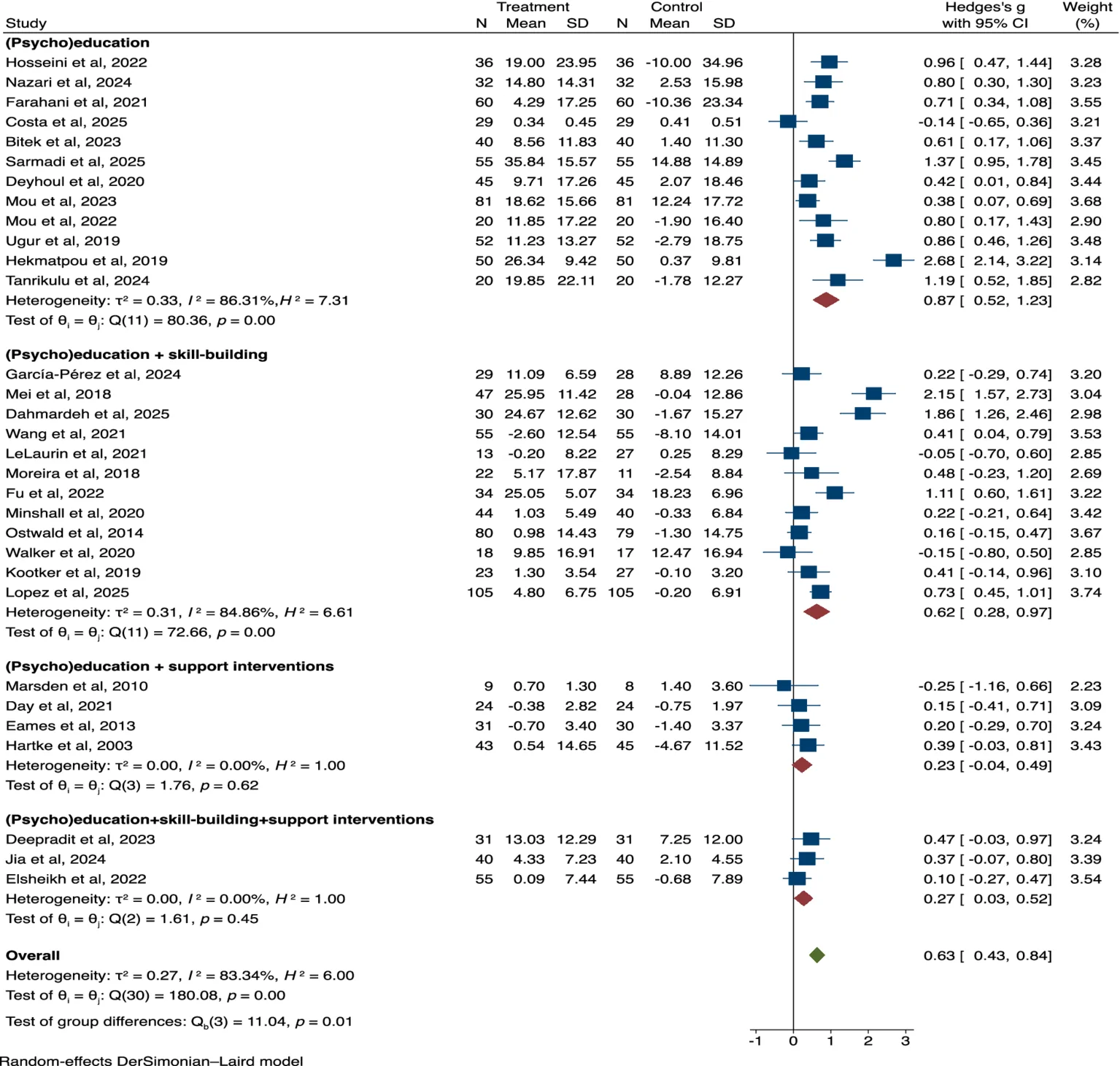
Meta-analysis of intervention types and caregiver burden of stroke informal caregivers.

#### 3.5.2 QoL of informal caregivers

The random-effects meta-analysis of 15 studies indicated that (psycho)education alone was significantly associated with improvements in informal caregivers’ QoL (*k* = 7; Hedges’ g = 1.11; 95% CI: 0.39 to 1.83; *I^2^* = 94.09%). Conversely, no significant improvements were found when (psycho)education was combined with skill-building (*k* = 5; Hedges’ g = 0.71; 95% CI: −0.38 to 1.80; *I^2^* = 95.43%) or with support interventions (*k* = 3; Hedges’ g = −0.15; 95% CI: −1.16 to 0.86; *I^2^* = 91.51%) (Figure 3).

**Figure 3:**
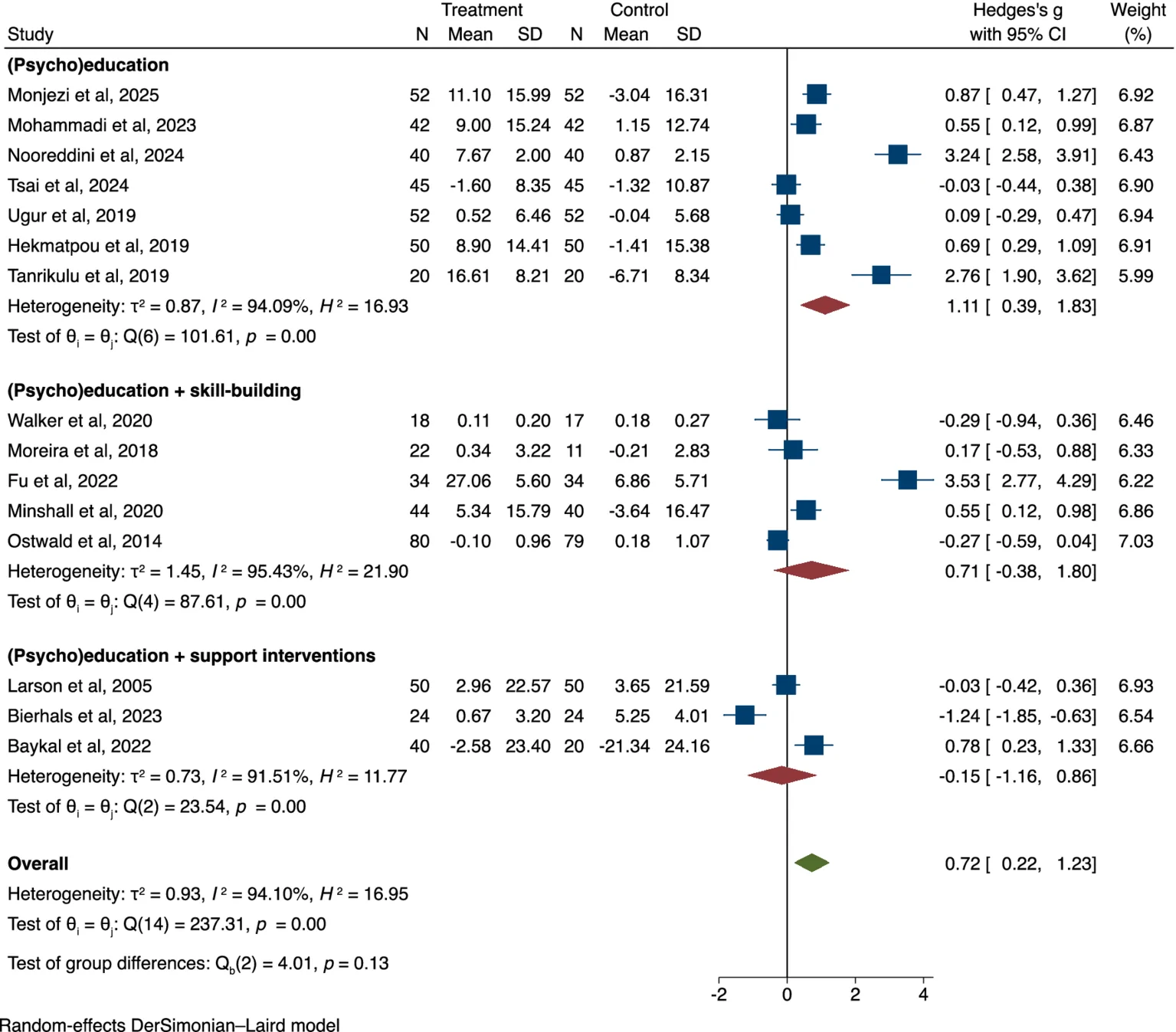
Meta-analysis of intervention types and quality of life of stroke informal caregivers.

### 3.6 Subgroup analyses

#### 3.6.1 Caregiver burden

Table 2 shows that no significant differences were observed across subgroups (*p* > 0.05 for all cases). However, although statistical significance was not reached, greater reductions in caregiver burden appeared to be noted in studies without theoretical frameworks (*k* = 14; Hedges’ g = 0.72; 95% CI: 0.29 to 1.15; *I^2^* = 89.63%) compared to theory-based studies (*k* = 18; Hedges’ g = 0.54; 95% CI: 0.36 to 0.72; *I^2^* = 66.55%). Interventions targeting informal caregivers alone tended to yield a greater reduction in caregiver burden (*k* = 15; Hedges’ g = 0.71; 95% CI: 0.39 to1.03; *I^2^* = 86.26%) compared to dyadic interventions (*k* = 17; Hedges’ g = 0.55; 95% CI: 0.30 to 0.80; *I^2^* = 79.19%). Larger reductions appeared to be associated with interventions designed by a mixed approach (*k* = 8; Hedges’ g = 0.83; 95% CI: 0.30 to 1.36; *I^2^* = 91.41%), delivered by researchers (*k* = 12; Hedges’ g = 0.83; 95% CI: 0.51 to 1.15; *I^2^* = 85.56%), implemented across both hospital and home settings (*k* = 13; Hedges’ g = 0.83; 95% CI: 0.47 to 1.19; *I^2^* = 87.91%), combining individual and group-based formats (*k* = 10; Hedges’ g = 0.72; 95% CI: 0.30 to 1.14; *I^2^* = 89.66%), using hybrid delivery modes (*k* = 12; Hedges’ g = 0.71; 95% CI: 0.32 to 1.10; *I^2^* = 87.94%), comprising 5*–*9 sessions (*k* = 21; Hedges’ g = 0.72; 95% CI: 0.43 to 1.01; *I^2^* = 87.01%), and lasting less than 2 months (*k* = 12; Hedges’ g = 0.73; 95% CI: 0.31 to 1.14; *I^2^* = 87.97%).

**Table 2:** Subgroup analysis of different intervention characteristics on caregiver burden (*N* = 32).

Intervention characteristics	*K*	*N*	SMD (95%CI)	*p* value for interaction	Heterogeneity	
					*I^2^* (%)	*P* value
Theoretical frameworks						
Theory-based (37, 39, 40, 42–44, 47, 50, 52, 55, 57–60, 69–72)	18	1,602	0.54 (0.36, 0.72)	0.439	66.55	< 0.001
Not theory-based (34–36, 48, 49, 53, 54, 56, 61–65, 68)	14	928	0.72 (0.29, 1.15)		89.63	< 0.001
Target population of interventions						
Stroke caregivers (34–36, 50, 52, 54, 55, 57, 58, 61, 63, 65, 68, 70, 71)	15	1,239	0.71 (0.39, 1.03)	0.435	86.26	< 0.001
Stroke survivor-caregiver dyads (37, 39, 40, 42–44, 47–49, 53, 56, 59, 60, 62, 64, 69, 72)	17	1,291	0.55 (0.30, 0.80)		79.19	< 0.001
Design of intervention						
One size fits all (34–36, 42, 48, 49, 60, 65, 68)	9	656	0.58 (0.22, 0.95)	0.599	80.12	< 0.001
Tailored (40, 47, 50, 53, 56–59, 61–63, 69–72)	15	1,132	0.53 (0.29, 0.77)		73.67	< 0.001
Mixed (37, 39, 43, 44, 52, 54, 55, 64)	8	742	0.83 (0.30,1.36)		91.41	< 0.001
Implementers of intervention						
Researchers (34, 35, 37, 42, 52, 54, 60, 63, 65, 68, 71, 72)	12	1,145	0.83 (0.51, 1.15)	0.222	85.56	< 0.001
Healthcare professionals (36, 43, 44, 47, 48, 50, 53, 56, 58, 59, 61, 62, 69)	13	845	0.45 (0.16, 0.74)		76.78	< 0.001
Multidisciplinary teams (39, 40, 49, 55, 57, 64, 70)	7	540	0.57 (0.09, 1.04)		85.06	< 0.001
Sites of intervention						
Hospital (49, 60, 61, 69)	4	190	0.36 (0.08, 0.65)	0.139	0.00	0.576
Home (34, 36, 37, 40, 42, 48, 50, 53, 55, 57, 58, 62, 64, 71, 72)	15	1,258	0.52 (0.26, 0.79)		80.49	< 0.001
Hospital + home (35, 39, 43, 44, 47, 52, 54, 56, 59, 63, 65, 68, 70)	13	1,082	0.83 (0.47, 1.19)		87.91	< 0.001
Delivery format of intervention						
Group (42, 49, 55, 60)	4	259	0.44 (0.14, 0.74)	0.521	29.08	0.238
Individual (34, 37, 40, 47, 48, 50, 53, 56, 57, 59, 61–64, 69–72)	18	1,324	0.62 (0.36, 0.87)		80.71	< 0.001
Combined (35, 36, 39, 43, 44, 52, 54, 58, 65, 68)	10	947	0.72 (0.30, 1.14)		89.66	< 0.001
Delivery modes of intervention						
Face-to-face (34–37, 40, 48, 49, 57, 60–63, 68)	13	984	0.64 (0.31,0.97)	0.556	83.44	< 0.001
Remote (42, 50, 52, 53, 55, 64, 71)	7	612	0.47 (0.18,0.75)		64.45	0.010
Hybrid (39, 43, 44, 47, 54, 56, 58, 59, 65, 69, 70, 72)	12	934	0.71 (0.32,1.10)		87.94	< 0.001
No. of intervention sessions						
0–4 (53, 60, 62)	3	210	0.52 (0.08, 0.96)	0.172	60.39	0.080
5–9 (36, 37, 39, 43, 44, 47–50, 52, 54–57, 59, 63–65, 70–72)	21	1,609	0.72 (0.43, 1.01)		87.01	< 0.001
≥ 10 (34, 35, 40, 42, 58, 61, 68, 69)	8	711	0.39 (0.21, 0.58)		31.17	0.179
Duration of intervention						
< 2 months (36, 42–44, 49, 53, 54, 56, 60, 62, 63, 68)	12	863	0.73 (0.31, 1.14)	0.502	87.97	< 0.001
2–6 months (34, 37, 39, 40, 47, 48, 50, 52, 55, 57–59, 61, 64, 65, 69–72)	19	1,557	0.57 (0.34, 0.79)		79.02	< 0.001
> 6 months (35)	1	110	0.41 (0.04, 0.79)			

*K*, number of studies; *N*, sample size.

#### 3.6.2 QoL of informal caregivers

As shown in Table 3, significant differences in effect sizes were observed among the subgroups categorized by theoretical framework, intervention designs, and intervention delivery modes (*p* < 0.05). Specifically, compared with studies that were not theory-based (*k* = 8; Hedges’ g = 0.88; 95% CI: 0.26 to 1.49; *I^2^* = 81.90%), theory-based studies were significantly associated with improvements in informal caregivers’ QoL (*k* = 8; Hedges’ g = 1.30; 95% CI: 0.47 to 2.14; *I^2^* = 96.31%). Greater effect sizes were noted for tailored interventions (*k* = 11; Hedges’ g = 0.91; 95% CI: 0.21 to 1.62; *I^2^* = 95.57%) compared to one-size-fits-all interventions (*k* = 2; Hedges’ g = −0.10; 95% CI: −0.43 to 0.24; *I^2^* = 0.00%) or mixed-design interventions (*k* = 3; Hedges’ g = 0.44; 95% CI: 0.07 to 0.80; *I^2^* = 59.29%). Similar patterns were also found for hybrid delivery modalities (*k* = 7; Hedges’ g = 1.23; 95% CI: 0.53 to 1.93; *I^2^* = 93.25%) compared to face-to-face modalities (*k* = 8; Hedges’ g = 0.21; 95% CI: −0.44 to 0.85; *I^2^* = 93.25%). Nevertheless, although no significant differences were detected in the other subgroup analyses, greater improvements in QoL were observed when interventions were delivered exclusively to informal caregivers (*k* = 9; Hedges’ g = 0.88; 95% CI: 0.26 to 1.49; *I^2^* = 92.77%), delivered by researchers (*k* = 5; Hedges’ g = 0.58; 95% CI: 0.30 to 0.87; *I^2^* = 56.05%), implemented across both hospital and home settings (*k* = 4; Hedges’ g = 1.78; 95% CI: 0.52 to 3.03; *I^2^* = 95.32%), delivered individually (*k* = 11; Hedges’ g = 0.92; 95% CI: 0.22 to 1.62; *I^2^* = 95.55%), comprised 5*–*9 sessions (*k* = 7; Hedges’ g = 0.99; 95% CI: 0.20 to 1.78; *I^2^* = 94.34%), and lasted 2*–*6 months (*k* = 10; Hedges’ g = 0.75; 95% CI: 0.21 to 1.30; *I^2^* = 92.85%).

**Table 3:** Subgroup analysis of different intervention characteristics on quality of life of stroke informal caregivers (*N* = 16).

Intervention characteristics	*K*	*N*	SMD (95%CI)	*p* value for interaction	Heterogeneity	
					*I^2^* (%)	*p* value
Theoretical frameworks						
Theory-based (37, 40, 41, 45, 47, 57, 58, 70)	8	729	1.30 (0.47, 2.14)	0.011	96.31	< 0.001
No theory-based (36, 38, 46, 51, 54, 61, 66, 67)	8	550	0.10 (−0.31, 0.51)		81.90	< 0.001
Target population of interventions						
Stroke caregivers (36, 45, 46, 51, 54, 57, 58, 61, 70)	9	674	0.88 (0.26, 1.49)	0.368	92.77	< 0.001
Stroke survivor-caregiver dyads (37, 38, 40, 41, 47, 66, 67)	7	605	0.43 (−0.33, 1.18)		94.98	< 0.001
Design of intervention						
One size fits all (36, 51)	2	135	−0.10 (−0.43, 0.24)	0.013	0.00	0.505
Tailored (38, 40, 41, 45, 47, 57, 58, 61, 66, 67, 70)	11	856	0.91 (0.21, 1.62)		95.57	< 0.001
Mixed (37, 46, 54)	3	288	0.44 (0.07,0.80)		59.29	0.086
Implementers of intervention						
Researchers (37, 45, 46, 54, 67)	5	452	0.58 (0.30, 0.87)	0.436	56.05	0.059
Healthcare professionals (36, 38, 41, 47, 51, 58, 61, 66)	8	560	0.30 (−0.40, 0.99)		93.64	< 0.001
Multidisciplinary teams (40, 57, 70)	3	267	1.99 (−0.72, 4.69)		98.22	< 0.001
Sites of intervention						
Hospital (38, 45, 51, 61, 67)	5	387	0.35 (−0.07, 0.77)	0.099	75.10	0.003
Home (36, 37, 40, 46, 57, 58, 66)	7	608	0.32 (−0.41, 1.05)		94.48	< 0.001
Hospital + home (41, 47, 54, 70)	4	284	1.78 (0.52, 3.03)		95.32	< 0.001
Delivery format of intervention						
Group (51)	1	100	−0.03 (−0.42, 0.36)	0.130		
Individual (37, 38, 40, 41, 45, 47, 57, 61, 66, 67, 70)	11	850	0.92 (0.22, 1.62)		95.55	< 0.001
Combined (36, 46, 54, 58)	4	329	0.29 (−0.11, 0.69)		68.70	0.022
Delivery modes of intervention						
Face-to-face (36–38, 40, 51, 57, 61, 66)	8	637	0.21 (−0.44, 0.85)	0.036	93.25	< 0.001
Remote (46)	1	84	0.55 (0.12, 0.99)			
Hybrid (41, 45, 47, 54, 58, 67, 70)	7	558	1.23 (0.53, 1.93)		93.25	< 0.001
No. of intervention sessions						
0–4 (38, 41, 45, 66, 67)	5	382	0.72 (−0.45, 1.89)	0.106	96.27	< 0.001
5–9 (36, 37, 47, 51, 554 57, 70)	7	511	0.99 (0.20, 1.78)		94.34	< 0.001
≥ 10 (40, 46, 58, 61)	4	386	0.10 (−0.28, 0.48)		68.44	0.023
Duration of intervention						
< 2 months (36, 38, 41, 45, 54, 66)	6	457	0.54 (−0.42, 1.49)	0.697	95.58	< 0.001
2–6 months (37, 40, 46, 47, 51, 57, 58, 61, 67, 70)	10	822	0.75 (0.21, 1.30)		92.85	< 0.001

*K*, number of studies; *N*, sample size.

### 3.7 Publication bias, sensitivity analyses, and meta-regression analyses

Funnel plots and Egger’s tests indicated no evidence of publication bias for either (psycho)education alone or (psycho)education plus skill building (Supplementary Figures S2, S3, S5, S6). Leave-one-out sensitivity analyses corroborated the robustness of the meta-analysis results (Supplementary Figures S4, S7). Sensitivity analysis accounting for the clustered design of the single included cluster RCT (72) confirmed the stability of the pooled effect estimate for caregiver burden (adjusted Hedges’ g = 0.26; 95% CI: 0.00 to 0.51 vs. original Hedges’ g = 0.27; 95% CI: 0.03 to 0.53). Furthermore, sensitivity analyses excluding studies with a high risk of bias demonstrated largely comparable overall findings, with standalone (psycho)education consistently yielded the largest effect estimates for both caregiver burden and QoL of informal caregivers (Supplementary Figures S8, S9). Meta-regression analyses showed that mean caregiver age was not significantly associated with effect size in either the (psycho)education interventions (coefficient = −0.028; 95% CI: −0.133 to 0.078) or (psycho)education plus skill building interventions (coefficient = −0.016; 95% CI: −0.057 to 0.025). Intervention duration demonstrated similar findings on (psycho)education (coefficient = −0.032; 95% CI: −0.360 to 0.295) or (psycho)education plus skill building (coefficient = −0.048; 95% CI: −0.202 to 0.105).

## 4 Discussion

To our knowledge, this is the first systematic review and meta-analysis to provide comprehensive and comparable evidence of how different interventions and their characteristics affect caregiver burden and QoL of informal stroke caregivers. Our findings indicated that standalone (psycho)education tended to exhibit greater reductions in caregiver burden compared to (psycho)education combined with skill building or interventions combining (psycho)education, skill building, and support interventions. In contrast, (psycho)education combined with support interventions showed no significant association with reduced caregiver burden. Similarly, standalone (psycho)education was the only intervention that was significantly associated with improvements in informal caregivers’ QoL. Subgroup analyses revealed larger effect sizes for both outcomes when interventions targeted caregivers alone, were researcher-led, used hybrid delivery modes, spanned hospital and home settings, and comprised 5–9 sessions. However, small sample sizes across most included studies and substantial between-study heterogeneity necessitate cautious interpretation of these effect magnitudes.

Our study underscored the significant effects of standalone (psycho)education on reduced caregiver burden and improved QoL of informal caregivers. However, interventions with more components tended to show smaller reductions in caregiver burden. Several plausible explanations may account for these observations. Standalone (psycho)education may provide caregivers with essential knowledge and enhance their caregiving competence (21, 73), enabling them to manage stroke survivors’ care more effectively and support their recovery, which may in turn improve caregiver outcomes. However, multicomponent interventions may inadvertently introduce an excessive informational burden on caregivers. This could conceivably leave them feeling overwhelmed and potentially limit intervention benefits, particularly given that many caregivers are older individuals with a limited educational background (48, 59). These findings underscore the need for future empirical research to confirm these tentative explanations and evaluate the feasibility and acceptability of intervention implementation. Notably, the present review differs from previous systematic reviews that did not differentiate between specific intervention types or evaluate their comparative effects on caregiver outcomes (18, 25). For instance, a review by Zhang et al. grouped heterogeneous interventions under the broad category of psychosocial interventions and found no significant effects on caregiver burden and QoL (19), making it difficult to determine which specific intervention types were associated with beneficial effects. Another review systematically categorized different intervention types but did not perform a meta-analysis to quantify the effects of each intervention category (26). By addressing these two gaps, our review provides more nuanced quantitative evidence to inform the implementation of caregiver interventions for stroke survivors.

Our subgroup analyses indicated that hybrid delivery modalities tended to show greater reductions in caregiver burden and larger improvements in caregivers’ QoL compared with face-to-face or remote interventions alone, aligning with previous reviews that suggested maximizing their synergistic strengths (17, 25). The face-to-face approach facilitates tailored emotional support and feedback, and caregiving experience sharing (74, 75), whereas remote methods enhance flexibility and accessibility (22, 23). The hybrid interventions may address key drivers of caregiver burden, such as time constraints and conflicting responsibilities (14), thereby contributing to improved caregiver QoL. Similarly, interventions spanning hospital and home settings showed larger effects than single-setting interventions, highlighting the importance of support continuity across the stroke care pathway. Inpatient-only interventions may inadequately support the transition to home and long-term recovery after discharge, whereas postdischarge-only interventions may miss the early caregiver education and preparation. Consistent with prior evidence (76), these findings underscore the necessity of continuous caregiver support bridging pre-discharge preparation and home-based recovery.

Our subgroup analyses also showed that interventions targeting caregivers alone appeared to yield larger effect estimates for reducing caregiver burden and improving caregiver QoL than dyadic interventions, which aligns with recommendations from the American Heart Association and American Stroke Association (77). Caregiver-focused interventions could address caregivers’ unique needs, whereas dyadic interventions may prioritize patient care and dilute caregiver support. Additionally, researcher-led interventions demonstrated more favorable outcomes than healthcare professional-or-multidisciplinary-led interventions, possibly reflecting higher intervention fidelity. Regarding session dosage, our findings aligned with previous reviews favoring 5–9 sessions (26, 77). A possible explanation is that fewer sessions may lack sufficient exposure to essential information, whereas overly intensive sessions may increase caregiver burden and undermine adherence (77). Notably, although subgroup differences lacked statistical significance, interventions without theoretical frameworks yielded numerically greater reductions in caregiver burden than the theory-based interventions, whereas the opposite pattern was observed for caregiver QoL. A possible interpretation is that non-theoretical interventions may prioritize pragmatic, task-oriented skill acquisition, thereby alleviating daily caregiving strain. Conversely, by systematically addressing cognitive restructuring, emotional regulation, and coping mechanisms, theory-based interventions may better enhance caregiver QoL as a complex, multidimensional outcome. Nonetheless, these exploratory subgroup analyses examined intervention characteristics separately and, therefore, could not determine an optimal intervention package, underscoring the need for future research. Furthermore, while our results demonstrated overall positive effects, the substantial heterogeneity observed across the synthesized evidence remains unexplained, which necessitates cautious interpretation and may limit the strength and generalizability of these findings.

### 4.1 Limitations

There are several limitations to this study. First, substantial heterogeneity was detected among the included studies. Although we performed meta-regression analyses by mean caregiver age and intervention duration, heterogeneity was not adequately explained. Future research should investigate whether this can be attributed to study or population characteristics, such as caregiver sex, caregiving relationship, and outcome assessment tools. Second, patient-level data were insufficiently reported among the included studies, limiting our ability to explore whether patient characteristics may influence caregiver outcomes. For example, time since stroke was not evaluated as a potential moderator. Given that caregivers’ needs may vary across the post-stroke continuum, future research should explore stage-tailored interventions. Third, caregiver-level social determinants, such as educational level and socioeconomic status, were sparsely or inconsistently reported across the included studies, precluding the evaluation of their potential moderating effects. Fourth, the effect estimates from some subgroup analyses should be interpreted with caution owing to the small number of studies in several subgroups and the predominance of small-sample trials. Finally, study omission remains possible owing to potential search deficiencies and English-language restrictions, despite a rigorous and extensive literature search.

## 5 Conclusion

Given the growing number of stroke survivors, informal caregivers have become increasingly central to post-stroke care and long-term recovery. This systematic review and meta-analysis showed that standalone (psycho)education has promise for reducing caregiver burden and improving the QoL of informal caregivers. Interventions with moderate intensity, using hybrid delivery, and spanning hospital and home settings had greater benefits in terms of caregiver outcomes. These findings have important clinical and practical implications for healthcare professionals, highlighting the need to integrate structured and continuous caregiver support across stroke care pathways. Larger methodologically rigorous studies are warranted to confirm this emerging evidence.

## Data Availability

The datasets presented in this study can be found in online repositories. The names of the repository/repositories and accession number(s) can be found in the article/Supplementary material.
