# Supplementary material for: Effectiveness of interventions for caregiver burden and quality of life of informal caregivers of stroke survivors: a systematic review and meta-analysis
Source: Front Public Health. 2026 Oct 7;14:1932478. doi: 10.3389/fpubh.2026.1932478 (PMC13683684; doi:10.3389/fpubh.2026.1932478)
Supplement: Supplementary file 1 [file Data_Sheet_1.docx]

Supplementary Material

# 1 Supplementary Data

Please refer to the Excel file in the uploaded attachments.

# 2 Supplementary Figures and Tables

## 2.1 Supplementary tables

**Table S1 Search strategy**

| **Database** | **No** | **Search strategy** |
| --- | --- | --- |
| **PubMed**  (386) | #1 | (((((((("Stroke"[Mesh]) OR ("Cerebrovascular Disorders"[Mesh])) OR ("Brain Ischemia"[Mesh])) OR ("Brain Infarction"[Mesh])) OR ("Cerebral Hemorrhage"[Mesh])) OR ("Subarachnoid Hemorrhage"[Mesh])) OR ("Stroke*"[Title/Abstract] OR "Cerebrovascular Accident*"[Title/Abstract] OR "Cerebral Stroke*"[Title/Abstract] OR "Stroke*, Cerebral"[Title/Abstract] OR "Cerebrovascular Apoplexy"[Title/Abstract] OR "Apoplexy, Cerebrovascular"[Title/Abstract] OR "Vascular Accident*, Brain"[Title/Abstract] OR "Brain Vascular Accident*"[Title/Abstract] OR "Cerebrovascular Stroke*"[Title/Abstract] OR "Stroke*, Cerebrovascular"[Title/Abstract] OR "Apoplexy"[Title/Abstract] OR "CVA (Cerebrovascular Accident)"[Title/Abstract] OR "Stroke*, Acute"[Title/Abstract] OR "Acute Stroke*"[Title/Abstract] OR "Cerebrovascular Accident*, Acute"[Title/Abstract] OR "Acute Cerebrovascular Accident*"[Title/Abstract])) OR (CVAs (Cerebrovascular Accident[Title/Abstract]))) OR ("Poststroke" [Title/Abstract] OR "Cerebral Infarction"[Title/Abstract] OR "Cerebral Hemorrhage"[Title/Abstract] OR "Intracerebral Hemorrhage"[Title/Abstract] OR "Brain Ischemia*"[Title/Abstract] OR "Brain Infarction"[Title/Abstract] OR "Cerebrovascular Disorder*"[Title/Abstract] OR "Cerebrovascular Disease*"[Title/Abstract]) |
|  | #2 | ("Caregivers"[Mesh]) OR ("Caregiver*"[Title/Abstract] OR "Care Giver*"[Title/Abstract] OR "Carer*"[Title/Abstract] OR "Family Caregiver*"[Title/Abstract] OR "Caregiver*, Family"[Title/Abstract] OR "Spouse Caregiver*"[Title/Abstract] OR "Caregiver*, Spouse"[Title/Abstract] OR "Informal Caregiver*"[Title/Abstract] OR "Caregiver*, Informal"[Title/Abstract]) |
|  | #3 | ((("Caregiver Burden"[Mesh]) OR ("Burden*, Caregiver"[Title/Abstract] OR "Caregiver Burden*"[Title/Abstract] OR "Caregiver Stress*"[Title/Abstract] OR "Stress*, Caregiver"[Title/Abstract] OR "Caregiving Burnout*"[Title/Abstract] OR "Burnout, Caregiving"[Title/Abstract] OR "Care Giving Burden*"[Title/Abstract] OR "Care Burden*"[Title/Abstract] OR "Caregiving Stress*"[Title/Abstract] OR "Stress*, Caregiving"[Title/Abstract] OR "Burnout*, Caregiver"[Title/Abstract] OR "Caregiver Burnout*"[Title/Abstract] OR "Caregiver Exhaustion"[Title/Abstract])) OR (Burden, Care Giving[Title/Abstract])) OR (Exhaustion, Caregiver[Title/Abstract]) |
|  | #4 | ("Quality of Life"[Mesh]) OR ("Quality of Life"[Title/Abstract] OR "Life Quality"[Title/Abstract] OR "Health-Related Quality Of Life"[Title/Abstract] OR "Health Related Quality Of Life"[Title/Abstract] OR "HRQOL"[Title/Abstract]) |
|  | #5 | ("Randomized Controlled Trial" [Publication Type]) OR ("Randomised Controlled Trial"[Title/Abstract] OR "Randomized Controlled Trial"[Title/Abstract] OR "Clinical Trials, Randomized"[Title/Abstract] OR "Trials, Randomized Clinical"[Title/Abstract] OR "Controlled Clinical Trials, Randomized"[Title/Abstract] OR "Randomized"[Title/Abstract] OR "Randomised"[Title/Abstract] OR "Controlled"[Title/Abstract] OR "RCT"[Title/Abstract] OR "RCTs"[Title/Abstract] OR "Randomized Clinical Trial"[Title/Abstract]) |
|  | #6 | #1 AND #2 AND (#3 OR #4) AND #5 |

**Table S2 Inclusion and exclusion criteria**

| **PICOST** | **Inclusion criteria** | **Exclusion criteria** |
| --- | --- | --- |
| Population (P) | Informal caregivers of stroke survivors (family members providing unpaid primary care), regardless of the age of caregivers or stroke survivors. | Paid or professional caregivers of stroke survivors |
| Intervention (I) | Active intervention strategies targeting either caregivers or dyads. | Studies examining interventions targeting stroke survivors exclusively rather than caregivers or caregiver-patient dyads. |
| Comparison (C) | Usual care, waitlist control, or any passive interventions. | Studies without an appropriate control group. |
| Outcomes (O) | Caregiver burden and Quality of Life (QoL), evaluated as either primary or secondary outcomes. | Studies lacking complete baseline and post-intervention outcome data |
| Study design (S) | Randomized controlled trials (RCTs) or cluster RCTs. | Reviews, trial registries, conference abstracts, letters, protocols, notes, or books. |
| Time & Setting | Any stage across the post-stroke continuum; hospital or community-dwelling settings; published in English. | Articles in languages other than English |

## 2.2 Supplementary Figures

## Figure S1 Risk assessment of bias

# Figure S2 Funnel plot of caregiver burden for (psycho)education

# Figure S3 Egger test of caregiver burden for (psycho)education

# Figure S4 Sensitivity analysis of caregiver burden for (psycho)education

# Figure S5 Funnel plot of caregiver burden for (psycho)education + skill-building

# Figure S6 Egger test of caregiver burden for (psycho)education + skill-building

# Figure S7 Sensitivity analysis of caregiver burden for (psycho)education + skill-building

**Figure S8 Forest plot of sensitivity analysis for caregiver burden excluding studies with a high risk of bias**

**Figure S9 Forest plot of sensitivity analysis for quality of life excluding studies with a high risk of bias**
